# Developing a New Model of Interprofessional Education in Aging and IDD

**DOI:** 10.1093/geroni/igab046.869

**Published:** 2021-12-17

**Authors:** Phillip Clark, Faith Helm, Edward Ansello

**Affiliations:** 1 University of Rhode Island, Kingston, Rhode Island, United States; 2 Virginia Commonwealth University, Richmond, Virginia, United States

## Abstract

Health and social care providers are ill-equipped to address the complex needs of individuals growing older with IDD and their families when dementia is suspected or diagnosed. Addressing the growing need for professionals to acquire practical diagnostic, treatment, and management methods requires an interorganizational and interprofessional approach. A consortium of aging and IDD organizations developed a successful Project ECHO (Extension for Community Healthcare Outcomes) model to create a virtual community of practice connecting a hub team and participating spoke sites. This paper reviews reasons for the model’s success, including: (1) curriculum providing practical solutions to complex problems, (2) integration of interprofessional team approach, (3) “all teach, all learn” model promoting sharing among participants, and (4) the inclusion of case studies engaging participants in developing solutions and strategies to improve the quality of life of clients and families. Implications of this model and recommendations for future professional educational programs are presented.

